# Causative role of left aIPS in coding shared goals during human–avatar complementary joint actions

**DOI:** 10.1038/ncomms8544

**Published:** 2015-07-08

**Authors:** Lucia M. Sacheli, Matteo Candidi, Vanessa Era, Salvatore M. Aglioti

**Affiliations:** 1 Department of Psychology, University of Rome ‘Sapienza', Rome I-00185, Italy; 2 IRCCS, Fondazione Santa Lucia, Rome I-00179, Italy

## Abstract

Successful motor interactions require agents to anticipate what a partner is doing in order to predictively adjust their own movements. Although the neural underpinnings of the ability to predict others' action goals have been well explored during passive action observation, no study has yet clarified any critical neural substrate supporting interpersonal coordination during active, non-imitative (complementary) interactions. Here, we combine non-invasive inhibitory brain stimulation (continuous Theta Burst Stimulation) with a novel human–avatar interaction task to investigate a causal role for higher-order motor cortical regions in supporting the ability to predict and adapt to others' actions. We demonstrate that inhibition of left anterior intraparietal sulcus (aIPS), but not ventral premotor cortex, selectively impaired individuals' performance during complementary interactions. Thus, in addition to coding observed and executed action goals, aIPS is crucial in coding ‘shared goals', that is, integrating predictions about one's and others' complementary actions.

## Abstract (web summary)

The neural mechanisms supporting imitative motor interactions have been well studied. However, considerably less is known about the mechanisms supporting complementary interactions. Here the authors demonstrate a causal role for left anterior intraparietal sulcus in coding complementary motor goals.

## Graphical abstract

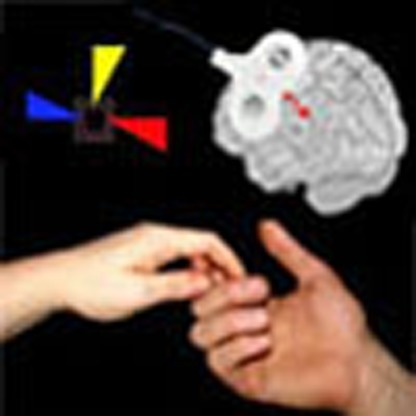

Understanding the behavioural mechanisms and neurological underpinnings of social interactions cannot be based on the exploration of the neural responses of a single person passively observing the behaviour of another. One critical epistemological and experimental challenge for the field of social neuroscience is to create dynamic contexts for the study of interpersonal behaviours without sacrificing well-controlled laboratory conditions^1^,^2^,^3^. This issue becomes crucial in the study of joint actions, defined as motor interactions in which two or more agents share a common goal^4^. In this domain, it is essential to devise interactive experimental paradigms, where the agent's goal is inherently linked to and dependent on that of a partner, suitable for exploring the neural correlates not only of imitative behaviours but also of non-imitative behaviours. Indeed, while imitation is adaptive in a variety of social circumstances, as during observational learning, and has been well investigated in terms of its neuro-cognitive bases, it turns out to be detrimental in several everyday dyadic encounters where complementary actions are necessary for achieving common goals. For instance, ‘doing what another individual is doing' may prove detrimental in some interpersonal encounters, for example, when dancing a tango where complementary moves need to be performed. Thus, the supposedly automatic^5^,^6^ and predictive^7^ sensory–motor simulation triggered by action observation reported by monkey and human neurophysiology^8^,^9^,^10^, neuropsychology^11^,^12^, behavioural^5^,^6^ and imaging studies^13^,^14^, might be only a component of the process supporting the ease with which we entertain skilful non-imitative motor interactions^15^,^16^,^17^,^18^. Although interpersonal coordination may benefit from the ability to predict a partner's action^19^, mere prediction of the others' action does not suffice in supporting, and may even interfere with, the implementation of appropriate complementary responses^17^. For instance, a tangoing lady needs to predict the next step of the leading partner to anticipate which complementary move she has to perform. Yet, the implementation of the complementary move needs to be decoupled from anticipatory simulation. It is thus important for the lady to shift from simulation to complementary adaptation while dancing.

Neuroimaging studies suggest that simulative processes occurring within the fronto–parietal network are recruited during the execution of both imitative and non-imitative (that is, complementary) interactions^20^, and that complementary actions might require additional neural resources to integrate and flexibly remap one's own actions with those of others^21^. However, no study has thus far explored whether any specific neural substrate may play a selective and causal role in mediating the execution of complementary motor interactions as compared with imitative ones.

Studying imitative and complementary interactions in the form of joint actions allows a dissociation of two crucial aspects of human motor behaviour, namely the prediction of others' movements^19^ and the representation of ‘shared goals'^22^. Indeed, during synchronous imitation, the agent's and partner's motor features overlap in terms of motor schemata, so that imitative interactions might benefit from pure anticipatory action–perception matching. On the contrary, complementary joint actions require, by definition, a spatial and/or temporal mismatch between one's own and others' movements and sub-goals. For instance, moving a table together (joint action) requires a mismatch between self-executed movements (for example, pushing the table, the motor sub-goal of one partner) and those observed in the other (for example, pulling the table, the sub-goal of the other partner). Without decoupling such movements and sub-goals, and controlling their integration, the shared goal of complementary joint actions cannot be achieved. Thus, complementary interactions possibly rely upon neural substrates dedicated to link and integrate self and others' movements and sub-goals in a single and coherent motor plan. Such coherent integration is here defined as a ‘shared goal' motor representation to prompt the idea that a joint action goal is both ‘in common' between co-agents and ‘divided up' into individual sub-goals that each actor needs to achieve to have the joint action fully accomplished. Please note that the definition of ‘shared goal' we refer to^22^ crucially differs from what philosophical reflections and empirical investigations on cooperation have defined ‘shared intentionality'^23^,^24^,^25^, which implies a commitment in achieving a state of affairs together with another agent rather than achieving that state alone. Stating that joint actions are characterized by shared goals suggests they are characterized by a ‘hierarchical structure' where the accomplishment of a (shared) overarching desired goal depends on the fulfilment of co-agents' sub-goals. Despite the importance of complementary joint actions for social life, information about which brain areas may causally and selectively support the implementation of shared goals during online complementary interactions is lacking.

To explore this issue, we used an offline continuous inhibitory Theta Burst Stimulation (cTBS) protocol through which we temporarily inhibited the neural activity of the targeted cortical areas. We tested whether two key nodes of the human fronto–parietal network, the left anterior intraparietal sulcus (aIPS) and the left ventral premotor area (vPM) play any active, crucial role in the execution of imitative and non-imitative reach-to-grasp movements of healthy participants interacting with a virtual partner. Notably, left aIPS has been widely associated with both goal-based motor control during movement execution^26^, and with the coding of others' observed action goals^27^,^28^, thus becoming a good candidate to be the neural substrate of shared goal representation during motor interactions.

In two different experiments, a total of 26 participants were asked to grasp a bottle-shaped object placed in front of them via either a precision or a power grip (that is, grasping the upper or the lower part of the bottle, respectively; see Methods). Participants were asked to perform their movements synchronously with a virtual partner shown on a screen in front of them. They were required to online adapt to the avatar's movements performing either the opposite (complementary) or the same (imitative) action (Fig. 1). We selected a grasping movement as its neurophysiological bases have been well explored during both actual execution^29^ and observation^30^, thus becoming an ‘experimental test-case' for goal-directed behaviours^31^. Importantly, it has also been shown that precision and power grasping are dissociable both under the neurophysiological and cognitive point of view, and can thus be considered different actions^29^,^31^.

To foster participants' need for online adaptation, in half of the trials, the virtual partner performed a movement correction during the reaching phase, shifting from power to precision grip (or vice versa). Individuals' accuracy and grasping asynchrony (GAsynchr; that is, the absolute time delay between the participant's and avatar's touch time on the bottle) were assessed as critical behavioural-dependent variables indexing the success of interpersonal coordination. Kinematics of both reaching (wrist trajectory indexed by wrist height) and hand pre-shaping (grip aperture indexed by index-thumb three-dimensional (3D) distance) components of participants' movements were monitored via high-sensitivity infrared cameras. This allowed us to test whether participants were actually online adapting to the avatar's movements. The joint-grasping task was preceded by a 20-s offline inhibitory continuous cTBS on either the left aIPS or the left vPM, and a sham stimulation on the vertex was included (experiment 1). To replicate results found in experiment 1, in experiment 2, real cTBS was applied over left aIPS or over the vertex as a control site (see Methods section). To discriminate the role of object affordances, we used identical hand–object interactions in imitative and complementary conditions. Thus, in imitative interactions, the avatar's and the individual's sub-goals are identical (for example, grasp the upper part of the bottle with a precision grip while predicting a precision grip from the avatar's movement), whereas complementary interactions required controlling the integration of two different sub-goals (for example, grasp the upper part of the bottle with a precision grip while predicting a power grip from the avatar's movement). We reasoned that finding a selective effect after the inhibition of left aIPS for the execution of complementary, but not imitative, actions would highlight the role of this area in integrating the individual's and partner's complementary movements and sub-goals. Human participants always acted as followers: they were required to adapt to the leading virtual partner who did not adaptively respond to the participant's movements because he played a pre-recorded action. Importantly, however, the task can be considered as interactive since each participant could not successfully select his/her own response without taking into account the virtual partner's action. More precisely, the participant's action goal fulfilment strictly depended on the ability to adapt to the virtual partner's action both in space and time.

Results from both experiments indicate that inhibition of aIPS selectively reduces the ability to synchronize with the partner only during complementary joint actions while leaving imitative action performance unchanged. Thus, aIPS seems to be selectively and causally involved in the integration of one's own and others' actions and sub-goals during complementary joint action.

## Results

### Experiment 1: (aIPS versus vPM versus sham)

In the first experiment, we examined the impact of stimulating aIPS and vPM using cTBS (Fig. 2). A sham condition was included. All significant results are reported in Table 1.

We first examined movement kinematics. An analysis of variance (ANOVA) on maximum wrist height (maxH) showed a significant action-type × clip-type × movement-type interaction (*F*(1,13)=10.9, *P*=0.006), indicating that participants realistically interacted with the avatar and online adapted to its movements, recruiting sensory–motor simulative processes (Fig. 3a). Indeed, the significant triple interaction on maxH indicates that in power grips (that is, when required to grasp the bottle in the lower position), participants followed a significantly higher trajectory in correction than in no-correction both in imitative and complementary actions (*P* values <0. 001, see also the highly significant clip-type × movement-type interaction (*F*(1,13)=105.8, *P*<0.001, all *post hoc* tests *P* values <0.001): this shows that during correction-power grips, participants were truly performing a movement correction, changing trajectory from a higher (that is, precision grip position) to a lower one (that is, power grip position) during the reaching phase. Moreover, in the no-correction condition, complementary-power and imitative-power grips significantly differed (*P*<0.001), indicating that participants' wrist trajectory was influenced by the virtual partner's trajectory: participants followed a higher trajectory if they had to grasp the lower part of the bottle-shaped object while coordinating with an avatar grasping the higher part (complementary condition). This suggests that participants recruited sensory–motor simulative resources in order to online coordinate with the virtual partner.

An ANOVA on maximum grip aperture (maxAp) showed a significant action-type × clip-type × movement-type interaction (*F*(1,13)=19.9, *P*<0.001; Fig. 3b). This interaction demonstrates that in precision grips, maxAp was significantly larger in corrections as compared with no-corrections for both imitative and complementary actions (all *P* values <0.001, see also the highly significant clip-type × movement-type interaction (*F*(1,13)=118.4, *P*<0.001, all *post hoc* tests *P* values <0.001): this indicates that participants pre-shaping shifted from a power to a precision grip during the reaching phase, online adapting to the avatar's movement correction. Moreover, the triple interaction indicates that maxAp in no-correction-precision grips during complementary actions were significantly larger than during imitative actions (*P*=0.001): this suggests pre-shaping was larger when participants had to perform a precision grip while interacting with the avatar performing a power grip (as a result of sensory–motor simulation of the avatar's movement).

None of the significant effects on maxH and maxAp was modulated by stimulation site (all *P* values >2). These analyses demonstrated that participants' kinematics was modulated both by the need to online correct one's own movements when the avatar performed a correction and by sensory–motor simulation triggered by the actions observed in the avatar, which induced visuo–motor interference in complementary actions where the avatar's movements are incongruent with the participant's ones.

We next examined joint coordination performance. An ANOVA on GAsynchr showed a significant main effect of action-type (*F*(1,13)=11.88, *P*=0.004). This effect was entirely qualified by the stimulation site (action-type × site, *F*(2,26)=5.37, *P*=0.011), because GAsynchr was higher (indicating a longer time delay between the participant's and the avatar's grasp times on the bottle, and thus a poorer performance) in complementary than imitative interactions only after inhibition of aIPS (*P*<0.001) and not after vPM or sham stimulation (*P*=0.14 and 0.63, respectively). Moreover, performance in complementary interactions after aIPS inhibition was significantly poorer (that is, GAsynchr was higher, indicating a longer time delay) than in all other conditions (all *P* values <0.015; Fig. 4a).

Finally, an ANOVA on GAsynchr also showed a significant action-type × clip-type × movement-type interaction (*F*(1,13)=89.37, *P*<0.001). *Post hoc* comparisons indicate that in the imitative-no-correction-precision grip condition, participants achieved a significantly higher level of performance with respect to all other conditions (all *P* values <0.003), and that in the imitative-no-correction-power grip condition there was an opposite trend (significantly lower performance, all *P* values <0.05 except as compared with complementary-no-correction-precision grips). Moreover, during complementary-correction condition, participants achieved lower performance as compared with complementary-no-correction condition for power grips, and better performance during precision grips. These effects show that the relative complexity of complementary versus imitative interactions depended on the combination with other conditions, and did not reflect a general difference between the two tasks. Crucially, these effects were not modulated by the stimulation site (site × action-type × clip-type × movement-type, *P*=0.7), and thus cannot account for results showed by the site × action-type significant interaction.

With regard to accuracy, Friedman ANOVA (see Methods section) gave no significant results in the comparison of interest (*χ*^2^(2)=2.97, *P*=0.22) indicating the lack of speed-accuracy trade-offs.

Finally, reaction times (RTs) showed a significant interaction between site and movement-type (*F*(2,26)=3.8, *P*=0.036); *post hoc* tests revealed that RTs after aIPS inhibition were faster than in all other conditions (all *P* values <0.025). However, this effect did not interact with action-type (site × action-type × movement-type, (*F*(2,26)=2.19, *P*=0.13), hence it does not explain the significant effect on GAsynchr. Moreover, RTs data also showed a significant site × action-type interaction (*F*(2,26)=3.5, *P*=0.045); however, *post hoc* tests showed no significant results (all *P* values >0.16), thus indicating no significant differences in RTs between complementary and imitative actions after aIPS inhibition (*P*=0.77). This pattern of results suggests that the significant effect of aIPS inhibition on GAsynchr could not be owing to a modulation of efficiency in individual movement preparation (that is, motor planning) or attentional factors. The analysis of movement times (MTs) showed the absence of significant main effects or interactions with stimulation site (Table 1).

### Experiment 2: aIPS versus vertex

In experiment 2, we examined the impact of stimulating aIPS or vertex using cTBS (Fig. 2). All significant results are reported in Table 2.

We first assessed the influence of cTBS on movement kinematics. An ANOVA on maxH showed a significant action-type × clip-type × movement-type interaction (*F*(1,11)=5.6, *P*=0.038; Fig. 5), indicating that participants online corrected their movement trajectory when the avatar made a movement correction, and that participants showed sensory–motor simulation of the avatar movements, as revealed by the interference between self-executed actions and those observed in the avatar in the complementary condition, that is, when the avatar's movements are incongruent with those of the participant. Indeed, the significant triple interaction on maxH indicates that while in no-correction participants showed a significantly lower trajectory in power grips (that is, when they grasped the lower part of the bottle-shaped object) than precision grips (that is, when they grasped the upper part, all *P* values <0.001), correction-power and correction-precision grips did not differ (*P*=0.1 and 0.2 in complementary and imitative actions, respectively). This shows that during correction-power grips, participants were truly performing a movement correction, changing trajectory from a higher (that is, precision grip position) to a lower one (that is, power grip position) during the reaching phase. Moreover, in the no-correction condition, complementary-power and imitative-power grips significantly differed (*P*=0.002), indicating that participants' wrist trajectory was influenced by the virtual partner's trajectory. Indeed, participants followed a higher trajectory when they had to grasp the lower part of the bottle-shaped object while coordinating with an avatar grasping the higher part (that is, in the complementary condition). This suggests that participants recruited sensory–motor simulative resources in order to online coordinate with the virtual partner.

Consistently with the effects emerged in maxH, the ANOVA on maxAp also showed a significant action-type × clip-type × movement-type interaction (*F*(1,11)=11.6, *P*=0.006; Supplementary Fig. 1). It indicates that maxAp in precision grips was significantly larger in corrections as compared with no-corrections for both imitative and complementary actions (all *P* values <0.001, see also the highly significant clip-type × movement-type interaction (*F*(1,11)=175.7, *P*<0.001, all *post hoc* tests *P* values <0.001), indicating that when participants watched the avatar performing a correction they online adapted to him by performing a movement correction. Moreover, maxAp in no-correction-precision grips during complementary actions was significantly larger than during imitative actions (*P*=0.001), indicating that it was larger when participants had to perform a precision grip while interacting with the avatar performing a power grip (as a result of sensory–motor simulation of the avatar's movement). As in experiment 1, none of the significant effects emerged in maxH or maxAp was modulated by stimulation site (all *P* values >0.5).

We next examined the impact of cTBS on joint coordination performance. An ANOVA on GAsynchr showed a significant main effect of clip-type (*F*(1,11)=14.69, *P*=0.003), indicating that coordinating with the avatar during the correction condition was overall more difficult than during trials in which the virtual partner did not correct its movements online. However, the higher-order action-type × clip-type × movement-type significant interaction (*F*(1,11)=6.14, *P*=0.031) indicated that the only significant difference in GAsynchr between corrections and no-corrections was found during imitative-precision grip (*P*=0.049). These effects were not modulated by stimulation site (in all interactions including the factors clip-type and site *P* values >0.3).

Regardless of the role of corrections, an ANOVA on GAsynchr showed a stimulation site (aIPS/vertex) × action-type (complementary/imitative) significant interaction (*F*(1,11)=7.54, *P*=0.019), confirming experiment 1 results. *Post hoc* tests showed that stimulation of left aIPS caused a selective decay of performance in complementary actions, so that after aIPS temporal inhibition joint coordination was significantly poorer (that is, GAsynchr was higher, indicating a longer time delay between the participant's and the avatar's grasp times on the bottle) during complementary as compared with imitative actions (*P*=0.02), and it also tended to be poorer as compared with complementary actions after vertex stimulation (*P*=0.06). On the contrary, complementary and imitative actions achieved an equal level of joint synchrony after cTBS on the control site (vertex; Fig. 4b), which is in line with previous literature^15^,^16^,^17^,^18^,^32^ and experiment 1 results. This suggests that at baseline complementary and imitative actions do not differ in terms of overall difficulty.

With regard to accuracy, the Wilcoxon-matched pair test (see Methods section) gave no significant results in the comparison of interest (*z*=0.88, *P*=0.37), indicating the lack of speed-accuracy trade-offs. The absence of significant main effects or interactions with stimulation Site in RTs and MTs (Table 2) shows that the effect described above were not due to attentional factors or non-selective impairment in movement execution.

Altogether, results from the analysis of kinematics in both experiment 1 and experiment 2 suggest that the processes called into play during human–human interaction in a task similar to the one adopted here (that is, mutual adjustment and sensory–motor simulation; see refs 17, 18, 32) are also recruited when participants interact with virtual characters if the action goal cannot be accomplished without taking the virtual partner's movements into account and online adapting to them. None of these effects showed significant interactions with stimulation site, indicating that aIPS stimulation did not imply impairment in motor execution.

Moreover, the results emerged from GAsynchr in both experiments 1 and 2 demonstrate that aIPS is causally involved in scaffolding non-imitative motor interactions. More specifically, aIPS inhibition selectively worsened performance synchrony in complementary interactions regardless of the specific movement features (either precision or power grips).

## Discussion

A variety of processes play a role in supporting the deceivingly simple human ability to coordinate with others, ranging from automatic entrainment to high-level planning mechanisms (for example, perspective-taking)^33^. Although realistic complementary interactions may involve somatosensory and motor simulation putatively based on the activity of the so-called ‘mirror neurons' system^20^,^21^,^34^, little is known about which neural resources specifically underpin these behaviours. Moreover, virtually, no study has addressed the issue of whether specific brain areas are causally involved in implementing the ability to shift from the mere simulation of another's action to performing complementary responses in online interactions.

In the present study, we addressed this issue by combining non-invasive interferential brain stimulation with a novel behavioural paradigm based on reach-to-grasp movements. We found that transient inhibition of left aIPS selectively reduces the ability to online coordinate with a partner performing complementary motor responses. Importantly, the kinematics of individuals' movements was not affected by stimulation of either left aIPS or left vPM, thus ruling out the presence of low-level motor impairments due to the stimulation of motor-related areas.

The neurophysiological bases of grasping movements are comparatively well known, thanks to studies in monkeys and human beings during actual execution^29^ and observation^30^. Studies indicate that both vPM and aIPS (as well as their homologues in monkeys, F5 and the anterior intraparietal area, AIP) deal with visuo–motor transformations and are the main cortical regions activated by the observation of grasping actions^30^ and by planning this type of movements^35^. Specifically, both regions are engaged in the predictive coding of observed actions based on the simulation of sensory–motor cues^36^. On the one hand, left vPM is responsible for simulating future postural and goal states along movement paths^37^,^38^, thus likely allowing one to monitor the deployment of observed actions^39^. On the other hand, left aIPS may play a specific role in predictively coding ones' own and other people's goals and intentions^27^,^28^. This region integrates spatial and perceptual features of target objects during grasping^40^, and it seems to play an important role when agents need to control individual reach-to-grasp movements for implementing intended goals^26^,^41^. aIPS neurons, for example, discharge well ahead of a planned hand action, suggesting that this region takes part in the motor implementation of abstract intentions^41^. Direct stimulation of inferior parietal but not premotor areas in human beings generates the conscious intention to move^42^. Moreover, parietal but not premotor damage reduces the anticipatory electrophysiological activity arising when healthy individuals expect others to move, indicating inferior parietal areas are involved in predictive coding of others' actions^43^. aIPS (and its monkey homologous AIP) is involved in the extraction of goal-related information from others' object-directed movements^27^,^28^,^44^,^45^. In particular, Freund^46^ suggested that while vPM is undoubtedly recruited during action observation, the parietal cortex is recruited whenever an action involves objects, emphasizing the significance of parietal cortex for goal-directed motor behaviour^46^. Finally, thanks to its dense anatomical^47^ and functional^48^,^49^ connections with vPM, aIPS is the ideal candidate for integrating information about the physical environment with motor predictions forwarded by vPM during planning of individual motor execution as well as during observation of others' actions^50^. Such a role might be crucial for joint action. Indeed, the time constraints to achieve online interpersonal coordination prevent agents from just passively react to others' behaviour and thus require reliable predictions about the outcomes of others' movements to efficiently adapt one's own behaviour accordingly^19^. Yet, these predictions regarding the outcome of the partner's actions (that is, about the partner's sub-goals) become beneficial to the joint action fulfilment only when integrated in the agent's motor plan^4^, that is, only when they are bound to the agent's own sub-goals to represent the interaction shared goal.

Our data suggest that both the partner's and the agent's action sub-goals might be coded in aIPS, and that this region underpins the process of integrating individual co-agent's sub-goals in one and the same motor representation. Indeed, aIPS inhibition impairs only complementary interactions, which is the condition where the cues regarding the partner's kinematics are misleading because the agent observes a movement but has to perform a different one. Planning appropriate movements in complementary interactions hence requires a specific focus on the action sub-goal, which in our experiment consists of understanding which movement the partner is going to perform, and thus which part of the bottle-shaped object he/she is going to grasp. On the contrary, synchronous imitation might also be achieved, thanks to pure anticipatory action–perception matching, as here ‘self' and ‘other' motor schemata overlap. Tellingly, pure predictive simulation does not suffice in the complementary condition, which instead necessarily requires self-other integration, a process that seems causally linked to aIPS.

In line with this interpretation, studies directly testing the neural underpinnings of ‘low-level' and ‘high-level' action goal representations in non-human primates showed that the majority of neurons in the parietal cortex (including AIP) are tuned to higher-order action goal coding^44^. Thus, parietal regions might be crucial when individual and observed action goals have to be combined in ‘higher-order' shared goals.

Previous neuroimaging studies^20^,^21^ have consistently reported increased activity of both aIPS and vPM during both imitative and complementary actions, supporting our hypothesis that a functional interplay between these areas is crucial during dyadic motor interaction. However, the temporal resolution and correlational nature of these studies does not allow teasing apart the role of frontal and parietal areas in joint action execution. The present repetitive transcranial magnetic stimulation (rTMS) study clearly indicates that temporary inhibition of aIPS causally and selectively affects the ability to coordinate with others during complementary interactions. Given the offline nature of our stimulation protocol, the interpretation of results has to keep into account the role of the targeted area, as well as of its connectivity with other nodes of the functional network it is embedded in^51^,^52^. More specifically, the most parsimonious interpretation of our results is that aIPS inhibition has a strong impact on the functioning of the fronto–parietal network recruited during complementary joint actions^20^,^21^. Importantly, the within-subject design used in this study allows us to rule out that our main effect is biased by individual differences in performing imitative versus complementary actions, or by interindividual differences in sensitivity to the cTBS^53^. Kinematics results also demonstrate that the selective interference on complementary interactions contingent upon inhibition of aIPS is not owing to changes in the ability to perform grasping movements *per se*. This may seem in contrast with previous TMS studies showing an impact of aIPS interference on grasping execution^26^,^54^. However, these previous studies show that the impact of aIPS stimulation on individual's kinematics is strictly time locked. For example, aIPS disruption actively modifies grasping (i) within 65 ms after the perturbation of the target orientation triggering different grasping movements with respect to the pre-programmed ones^26^, and (ii) only if applied at specific instants, namely during online movement corrections, but not earlier, during action planning^54^. The lack of time specificity of our offline cTBS protocol may explain why we did not find a specific effect on individual grasping kinematics.

It is worth noting that previous TMS studies tested whether mirror-like resonant systems are involved in simulating not only congruent but also incongruent movements with respect to an observed one^55^,^56^,^57^,^58^,^59^,^60^. To this aim, studies investigate whether visuo–motor training and associative learning^57^ can modulate visuo–motor interference effect^5^. It is still hotly debated whether such interference depends on visuo–motor associations in vPM^57^, on self-other distinction blurring^58^,^59^ or on overload/modulation of prefrontal control areas^60^. In any case, none of the aforementioned processes is essential in the present study, where self-other goal integration in aIPS becomes crucial instead. Indeed, at variance with visuo–motor interference paradigms, our task does not imply that participants must ignore the partner's movements or merely ‘respond' to them. Rather, our participants are required to act together with the virtual partner, and the requirement of being synchronous with and adapting to the partner (by performing an imitative or complementary action) cannot be achieved without taking into account the partner's action. Consequently, in our task, the inclusion of partner's movements in the participant's motor plan becomes more relevant than visuo–motor association. This would also explain why the role of aIPS in complementary actions may be more crucial than the role of vPM.

In sum, our paradigm gets at a core aspect of joint action, namely, the need to integrate the predicted movement of a partner in one's own motor plan and to share a common goal. Goal sharing is essential for motor interactions, as two people will never manage to coordinate, for instance, while moving a table from the kitchen to the living room, if they do not both want to have it moved there. More precisely, the shared goal is what links the partners' actions during the interaction (for example, one pulls and the other pushes the table because they both want the table moved there).

Inspired by the notion that moving together is much more than just ‘I move while I see you moving at the same time', our study contributes to research on the neuro-cognitive bases of joint actions by stepping forward from investigations on sensory–motor coupling, on one hand, and inhibition of automatic imitation on the other. Recent electrophysiological^61^,^62^ and neuroimaging^63^,^64^ studies introduced interactive scenarios for exploring imitation/synchronization and joint attention. Here we capitalized on these intuitions to focus on the issue of complementarity, and to explore how people anticipate what a partner is doing in order to predictively and complementarily adjust their own movement accordingly. Considering that these situations naturally occur in everyday life since young childhood^65^, it might be likely that they do not require higher-level cognitive control or mental state attribution but rather rely on goal-directed motor processes^22^, as the shared goal coding in left aIPS.

## Methods

### Participants

Twenty-six participants took part in the study (experiment 1:14 participants, 2 males, aged 24.6±4.0 years; experiment 2:12 participants, 4 males, aged 24.5±4.3 years). No participant performed in both experiments. All participants were right-handed as confirmed by the Standard Handedness Inventory^66^, reported normal or corrected-to-normal vision and were naive as to the purpose of the experiment.

The experimental protocol was approved by the ethics committee of the Fondazione Santa Lucia and was carried out in accordance with the ethical standards of the 1964 Declaration of Helsinki.

None of the participants had neurological, psychiatric or other medical problems, or any contraindication for TMS^67^. Participants gave their written informed consent to take part in the study, received a reimbursement for their participation and were debriefed as to the purpose of the study at the end of the experimental procedures. No discomfort or adverse effects to rTMS were reported or observed in any of the participants.

### Materials and task

Experimental stimuli and set-up as well as task instructions and procedures during the interactive task were identical in experiments 1 and 2.

**Figure 1:**
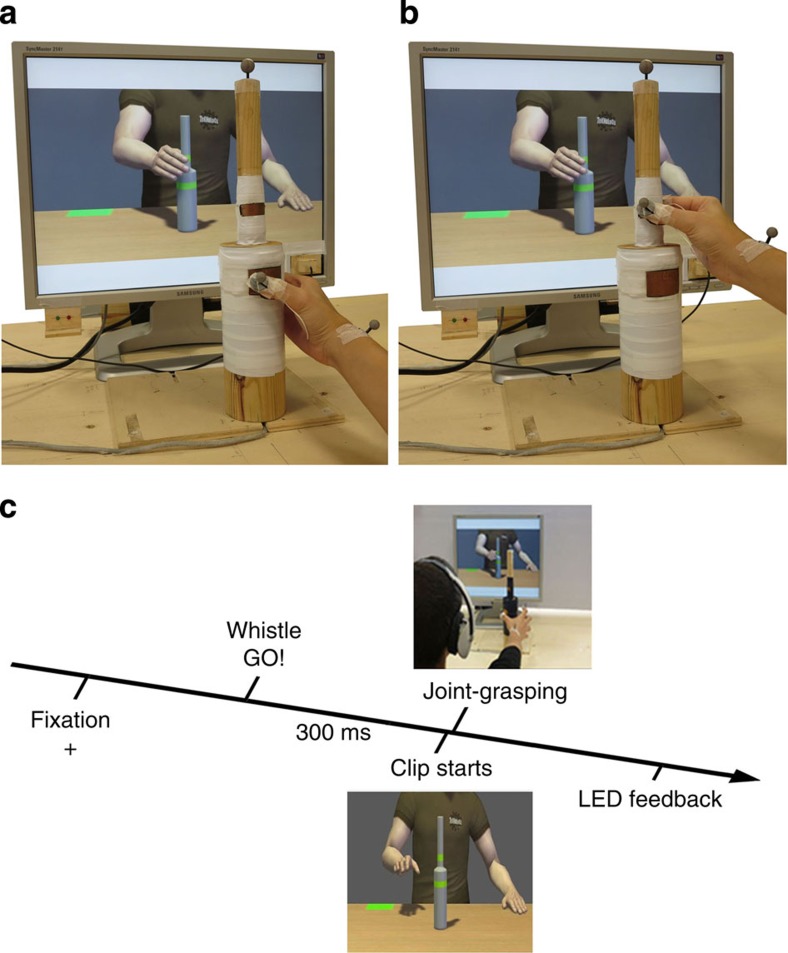
Pictures illustrating the experimental set-up from the participant's perspective and trial timeline. Participants sat in front of the screen and reached-to-grasp their bottle-shaped object trying to be as synchronous as possible with the avatar. Complementary (**a**) and imitative (**b**) actions are shown. A pair of green/red LED lights was placed on the right bottom corner of the screen in order to provide feedback signals about participants' performance. The photodiode to record the instant in which the avatar grasped the bottle-shaped object was placed on the left bottom corner of the screen. It is also possible to see the infrared reflective markers placed on the participant's right hand: kinematics has been recorded from the wrist (dorso–distal aspect of the radial styloid process), thumb (ulnar side of the nail) and index finger (radial side of the nail). (**c**) Trial timeline during the interaction task. Please note part of this figure has already been published in Fig. 1a in ref. 32.

*Experimental stimuli and set-up.* Participants were comfortably seated in front of a rectangular table of 120 × 100 cm and watched a 1,024 × 768 resolution LCD monitor placed on the table at a distance of ∼60 cm from their eyes. Participants had to reach and grasp a bottle-shaped object (30 cm total height) constituted by two superimposed cylinders with different diameters (small, 2.5; large, 7.0 cm) placed next to centre of the working surface, 45 cm away from the participants and 5 cm to the right of the midline. In order to record participants' touch time on the bottle, two pairs of touch-sensitive copper plates (one per each cylinder) were placed at 15 and 23 cm of the total height of the object. Before each trial, participants positioned their right hand on a starting button placed at a distance of 40 cm from the bottle-shaped object and 10 cm on the right of the midline, with their index finger and thumb gently opposed. The go-signal was delivered to participants via headphones (a whistle 4 db and 787.5 Hz). The feedback signals about participants' performance were provided via a green/red LED light placed next to the left corner of the screen (Fig. 1 and Supplementary Fig. 2).

Infrared reflective markers (5 mm diameter) were attached to participants' right upper limb on the following points: (i) thumb, ulnar side of the nail, (ii) index finger, radial side of the nail and (iii) wrist, dorso–distal aspect of the radial styloid process. Movement kinematics was recorded with a SMART-D motion capture system (Bioengineering Technology & Systems (B|T|S)). Four infrared cameras with wide-angle lenses (sampling rate 100 Hz) placed ∼100 cm away from each of the four corners of the table captured the movements of the markers in 3D space. The s.d. of the reconstruction error was always <0.5 mm for the three axes.

*Creation of virtual interaction partner and avatar's validation*. The kinematic features of the virtual partner were based on the movements of human participants actually performing different grasping movements during a human–human joint-grasping task identical to the one described in ref. 18. These grasping movements were performed with the right dominant hand and recorded using 3D motion capture procedures. Motion capture was performed using a Vicon MX optical tracking system (Vicon Motion Systems, Oxford, UK) with 10 infrared light-emitting cameras. The 3D positions of 37 passive reflecting markers, attached to the participant's complete upper body (pelvis, chest, head, left and right arm, and right hand) were recorded with a spatial error below 1.5 mm and at a temporal resolution of 120 Hz. Raw data were processed offline using commercial Vicon software to reconstruct and label the markers, and to interpolate short missing parts of the trajectories. The final processed trajectories were animated using commercial software (Autodesk, Motion Builder) in the appearances of a Caucasian male character. Since we wanted the participants to ignore facial expressions, the final video stimuli contained only the upper body down from the shoulders, without the neck and head (Fig. 1).

The complete sample of clips comprised 16 different grasping movements. Half of the grasping movements ended at the top position of the bottle-shaped object (that is, required precision grips), whereas the other half of the movements ended at the bottom position (that is, required power grips). Moreover, in 50% of the trials, the grasps included an online correction, in which the avatar performed a movement correction by switching from a precision to a power grip (or vice versa) during the reaching phase. Thus, the 16 clips could be divided in four conditions (correction/no-correction × power/precision grip), each comprising four different variants of the movement.

Before the interactive task, a pilot study was conducted in order to validate the movements of virtual character. Twelve participants, not included in the interactive tasks, were asked to rate ‘how natural and realistic' they perceived the avatar's movements to be on visual analogue scales ranging from 0 to 100. Participants' ratings indicate that the movements were perceived as realistic (63.8±24.4). More importantly, a two-way repeated measure ANOVA on the different types of clip (correction/no-correction × precision/power grip) indicated judgements did not show any significant difference (all *P* values >0.2).

*Interactive task*. Participants were required to perform the grasping task interacting with the same virtual partner. Namely, they had to reach and grasp with their right dominant hand the bottle-shaped object placed in front of them as synchronously as possible with the avatar (shown on a screen in front of them). Given the bottle-shaped object's dimensions, grasping the lower part would imply a whole-hand grasping (a power grip), whereas grasping the upper part would imply a thumb-index finger precision grip.

During each trial, participants had to adapt to the virtual partner's movement without knowing in advance which part of the bottle was to be grasped. Participants had to perform opposite/same movement with respect to their virtual partner in different blocks (that is, complementary versus imitative). More specifically, in imitative blocks, if the avatar was grasping the upper part of the object, participants also had to grasp the upper part. In contrast, in complementary blocks, participants had to perform the opposite movement with respect to the avatar (in this example, grasping the lower part of the object; Fig. 1). Both the participants' and the avatar's movements were performed with the right, dominant hand. Participants were instructed to grasp the object as synchronously as possible with the avatar in all conditions. Thus, the task required coordination in time (being synchronous) and space (doing complementary/imitative movements) between the participant and the avatar. The instruction to perform the opposite or same movement was provided on the screen at the beginning of each block.

The trial timeline was as follows: the presentation of each clip was preceded by a fixation cross placed on the region of the screen where the avatar's hand would appear. The cross had the purpose of alerting participants about the impending trial. Then, participants heard an auditory go-signal and (after 300 ms) the clip started. Upon receiving the auditory instruction, participants could release the start button and reach-to-grasp the bottle-shaped object. When participants started before hearing the instruction, the trial was classified as a false start and discarded from the analyses. At the end of each trial, participants received feedback (a green or red LED light turned on) about their performance (win/loss trial). A win trial required that participants followed their auditory instructions (that is, correctly performed complementary/imitative movements with respect to the avatar) and achieved synchrony in object grasping with the avatar. The action was considered synchronous when the time delay between the participant's and the avatar's index-thumb contact times on their bottle fell within a given time window which was narrowed or enlarged on a trial-by-trial basis according to a stair-case procedure. Thus, this procedure allowed tailoring the time window to set GAsynchr on the specific skill of each participant. In order to motivate individual commitment during the task, participants knew their monetary reward would depend on the number of wins accumulated during the experimental sessions. Note that the avatar's index-thumb contact times were measured trial-by-trial by a photodiode placed on the screen that sent a signal recorded by E-Prime2 software (Psychology Software Tools Inc., Pittsburgh, PA). The photodiode was triggered by a black dot displayed on the screen (but not visible to the participants) at the clip frame corresponding to the instant when the avatar grasped his virtual object. Before any brain stimulation and recording of the motor task, a familiarization block of four imitative and four complementary movements was delivered.

In each session (after cTBS), participants performed four 28-trial complementary/imitative blocks (in a counterbalanced order between participants). Since the clip sample comprised 16 clips divided in four conditions (correction/no-correction × power/precision grip, each including four different variants of the movement), in each block, three items (out of the four per condition) were repeated (final block-sample=7 items per condition, presented in randomized order). Both the imitative and complementary blocks were performed twice in a session. Thus, in each four-block session, participants performed 14 trials per condition (complementary/imitative × correction/no-correction × power/precision grip). Crucially, in imitative and complementary blocks, participants watched and adapted to exactly the same avatar's movements. Stimuli presentation and randomization were controlled by E-Prime2 software (Psychology Software Tools Inc.).

### Transcranial magnetic stimulation

The intensity of stimulation was determined for each participant relative to the participant's resting motor threshold (rMT). Participants wore a tightly fitting bathing cap on which scalp stimulation points were marked. Motor-evoked potentials (MEPs) were recorded from the first dorsal interosseous muscle of the right hand. Surface Ag/AgCl electrodes were placed in a belly-tendon montage with the active electrode placed over the motor point and the reference over the interphalangeal joint. Electromyographic recording was performed with a Viking IV (Nicolet Biomedical) electromyograph. The rMT, defined as the lowest intensity able to evoke 5 of 10 MEPs with an amplitude of at least 50 μV, was determined by holding the stimulation coil over the optimal scalp position. The optimal scalp position for inducing MEPs in the right first dorsal interosseous muscle was found by moving the coil in steps of 1 cm over the left primary motor cortex until the largest MEPs were found and then marked with a pen on a bathing cap worn by participants. Mean rMT was 57.6±7.9% of the stimulator output in experiment 1, and 59±7% of the stimulator output in experiment 2.

Stimulation sites were identified on each participant's scalp with the SofTaxic Navigator system (EMS). Skull landmarks (nasion, inion and two preauricular points) and 61 points providing a uniform representation of the scalp were digitized by means of a Polaris Vicra Optical Tracking System (NDI). Coordinates in Talairach space (Talairach and Tournoux, 1988) were automatically estimated by the SofTaxic Navigator from a magnetic resonance imaging (MRI)-constructed stereotaxic template using an individualized probabilistic head model computation. This individualized head model preserves the anatomical scalp–brain correlates of a mean magnetic resonance template, providing an accurate set of estimated MRI data, specific for the participant under examination. TMS was performed using a 70-mm figure-of-eight coil connected to a Magstim Super Rapid Transcranial Magnetic Stimulator (The Magstim Company). A 20-s cTBS paradigm was applied: it has been shown to have an inhibitory effect over the stimulated site starting from 5 min after stimulation and lasting up to 20 min after stimulation^68^.

The procedure was similar to Huang *et al.*^68^: trains of three pulses at 50 Hz were delivered every 200 ms (that is, at 5 Hz) for 20 s (300 pulses in total). cTBS was applied at 80% of the rMT (experiment 1, mean 46.2±4.7% of the stimulator output; experiment 2, mean 47±4.6% of the stimulator output). After the cTBS, participants rested for 5 min with their right arm relaxed on the side, then they started the interactive task. The task never lasted >10 min so that the inhibitory time window was never exceeded. Huang *et al.*^68^ reported that short (300 pulses) and long (600 pulses) cTBS protocols induce a comparable amount of inhibition. We adopted the short one here because it warranted that the inhibitory effect of cTBS had completely faded away 1 h after stimulation.

### Experiment 1

**Figure 2:**
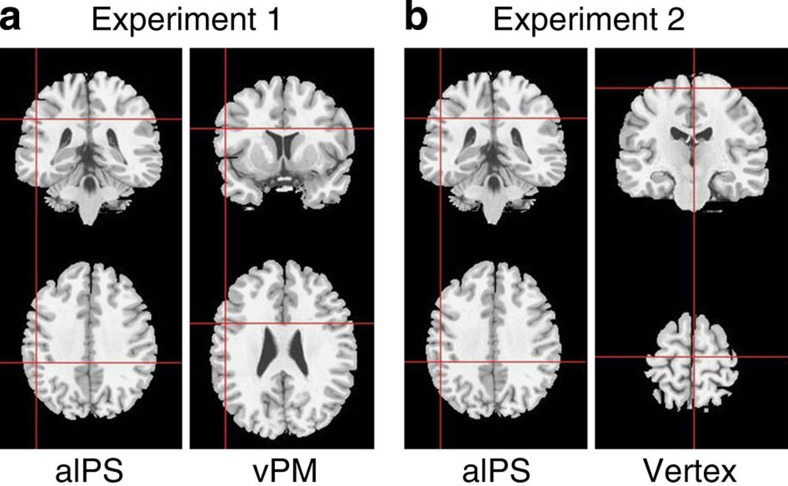
Mean stimulation sites in experiment 1 (*n*=14) and experiment 2 (*n*=12) reported on a brain template. (**a**) In experiment 1, 20 s offline inhibitory cTBS was applied over left aIPS (−48.7±1.08, −34.5±1.27 and 36.3±0.5 *Tal*) or left vPM cortex (−52.1±1.0, 10.12±1.6 and 23.5±0.6 *Tal*) as control site, and a sham stimulation was also used. (**b**) In experiment 2, cTBS was applied on either the left aIPS (−49.7±1.9, −34.5±1.6 and 36.1±0.5 *Tal*) or the vertex (3.6±0.8, −15.3±1 and 63.5±0.5 *Tal*) as control site.

**Figure 3:**
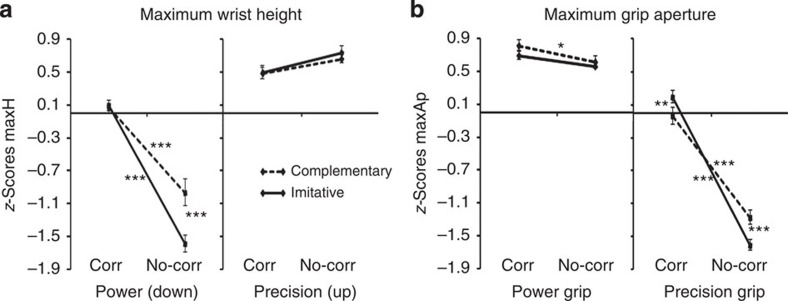
Kinematics data results from experiment 1 (*n*=14). These results indicate participants were truly adapting to the avatar's movements and recruited sensory–motor simulative processes during the task. ‘Corr', corrections; ‘No-corr', no-corrections. Error bars indicate s.e.m. ***P<0.001, **P<0.01 and *P<0.05. (**a**) Illustration of the action-type × clip-type × movement-type significant interaction (*F*(1,13)=10.9, *P*=0.006) emerged from the repeated measures ANOVA on maxH (*z*-scores mean values). For the sake of simplicity, we did not explicitly report in the figure the significance of the comparison between power and precision grips in all conditions (all *P* values ≤0.007, also indicated by the significant main effect of movement-type; Table 1). (**b**) Illustration of the action-type × clip-type × movement-type significant interaction (*F*(1,13)=19.9, *P*<0.001) emerged from the repeated measures ANOVA on maxAp (*z*-scores mean values). For the sake of simplicity, we did not explicitly report in the figure the significance of the comparison between power and precision grips in all conditions (all *P* values ≤0.001, also indicated by the significant main effect of movement-type; Table 1).

**Figure 4:**
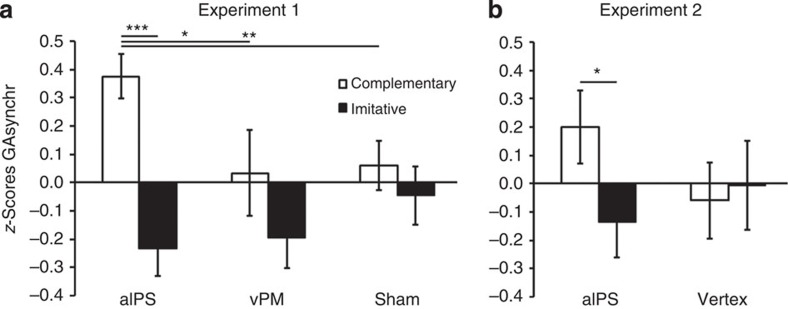
Joint coordination performance in terms of GAsynchr after cTBS stimulation in experiment 1 (*n*=14) and experiment 2 (*n*=12). Error bars indicate s.e.m. ***P<0.001, **P<0.01 and *P<0.05. In line with previous studies, participants achieved equally proficient performance in complementary and imitative actions in all control conditions (left vPM and vertex stimulation, and after sham stimulation). On the contrary, performance dissociated after left aIPS inhibition. (**a**) The figure illustrates the stimulation site (aIPS/vPM/sham) × action-type (complementary/imitative) significant interaction (*F*(2,26)=5.37, *P*=0.011) showed by the repeated measures ANOVA on GAsynchr *z*-scores in experiment 1. (**b**) The figure illustrates the site (aIPS/vertex) × action-type (complementary/imitative) significant interaction (*F*(1,11)=7.54, *P*=0.019) showed by the repeated measures ANOVA on GAsynchr *z*-scores in experiment 2.

**Figure 5:**
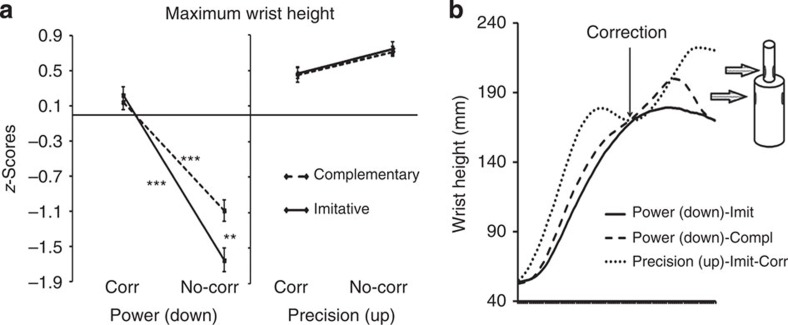
Kinematics data on maxH in experiment 2 (*n*=12). ‘Corr', corrections; ‘No-corr', no-corrections. (**a**) The figure illustrates the action-type × clip-type × movement-type significant interaction (*z*-score mean values) emerged from the repeated measures ANOVA on maxH. In no-corrections, participants showed a significantly lower trajectory when grasping the lower part of the bottle-shaped object (power grips) than when grasping the upper part (precision grips) (all *P* values <0.001, the significant difference is not reported in the graph for the sake of simplicity), while correction-power and correction-precision grips did not differ. This shows that during correction-power grips participants were truly performing a movement correction. Moreover, in no-corrections, complementary (Compl)-power and imitative (Imit)-power grips significantly differed, indicating that the participant's wrist trajectory was influenced by the virtual partner's one (see main text). The fact that these significant effects were more evident when participants grasped the lower part of the bottle-shaped object (that is, in power grips) is owing to the features of the recorded parameter (peak wrist height), implying a ceiling effect when participants correctly grasp the upper part of the bottle with a precision grip. Error bars indicate s.e.m. ***P<0.001, **P<0.01 and *P<0.05. (**b**) Raw wrist trajectory recorded from a representative participant during an imitative-power grip (thick line), complementary-power grip (striped line) and during a correction from power to precision grip (dotted line). The arrow indicates when the correction occurred in the wrist trajectory during the correction trial (in the example, the participant follows a lower trajectory as to grasp the lower part of the object and then she corrects to the trajectory aiming at the higher part). These data illustrate that (i) during complementary actions, participants are influenced by the movement observed in the avatar, for example, when grasping the bottle in the lower position they follow a higher trajectory simulating the avatar who is aiming at the higher position, and (ii) they online correct their own movements in trials when the avatar performs a correction, confirming they are online adapting to the partner during the task.

We used the SofTaxic Navigator system (EMS) in order to identify and store the sites that, according to the coordinates reported by Hamilton and Grafton^28^ (MNI −52, −32, 44, converted in *Tal* −47, −34, 37 according to ref. 50), were optimally targeting left aIPS. The same procedure was adopted for vPM coordinates (coordinates reported by Avenanti *et al.*^38^, *Tal* −52, 10, 24). Mean stimulation sites were 48.7±1.08, −34.5±1.27 and 36.3±0.5 for left aIPS and −52.1±1.0, 10.12±1.6 and 23.5±0.6 for left vPM (Talairach coordinates; Fig. 2a). During sham stimulation, TBS was delivered on a 3-cm-thick wooden rectangular-shaped object placed on the vertex of participants' head. Thanks to coil calibration, the navigation system allowed the overlap of the coil focus with these coordinates and made it possible to monitor online any movement of the coil during the 20-s cTBS. Displacements from the individual optimal scalp locations for aIPS/vPM stimulation never exceeded 2 mm for any of the three axes. aIPS/vPM/sham stimulation was counterbalanced between participants.

### Experiment 2

The scalp locations that allowed to optimally target left aIPS (as reported in experiment 1) and vertex coordinates (*Tal* 0, −17, 63; ref. 69) were identified and stored by the SofTaxic Navigator system (EMS). The resulting mean stimulation sites were −49.7±1.9, −34.5±1.6 and 36.1±0.5 for left aIPS, and 3.6±0.8, −15.3±1 and 63.5±0.5 for the vertex (Talairach coordinates; Fig. 2b). Displacements from the optimal individual scalp locations for aIPS/vertex stimulation never exceeded 2 mm for any of the three axes. aIPS/vertex stimulation was counterbalanced between participants.

### Data handling and analyses

Only correct trials were entered in the behavioural and kinematics analyses. More specifically, we excluded from the analyses trials in which participants (i) missed the touch-sensitive copper plates and response was thus not recorded; (ii) made false starts or (iii) did not respect their complementary/imitative instructions (on average, excluded trials=5.5±3.2% of total (18±11 trials) in experiment 1 and 6.4±2.3% of total (14±5 trials) in experiment 2).

We considered as crucial behavioural measures:

1. Accuracy, that is, percentage of movements executed according to the instructions (false starts were considered errors).
2. RTs, that is, time from the go-signal to the instant participants released the start button;
3. MTs, that is, time from the instant when participants released the start button to the instant their index and thumb contacted the bottle;
4. GAsynchr, that is, absolute value of time delay between the participant's and the avatar's index-thumb contact times on the bottle (abs(participant's contact time on the bottle minus the avatar's contact time on the bottle)); note that ‘contact time' is computed for both the participant and the avatar as the time from the go-signal onset to the instant their index and thumb touched the bottle.

For each of the above-mentioned behavioural measures, we calculated the individual mean in each condition. These values were entered in different within-subject ANOVAs (see below).

Moreover, we analysed kinematics associated to the reaching and pre-shaping component of the reach-to-grasp movement^70^ in order to monitor participants' motor execution during the task. Analysis of kinematics allowed us to ascertain that participants online adapted to the avatar's movements during the reach-to-grasp; this was done to verify that kinematics patterns reflected those in previous studies investigating human–human interactions using a similar set-up^17^,^18^,^32^. Moreover, we verified that TMS-induced inhibition did not generate an unspecific impairment in movement executions irrespective of the avatar's movements, that is, independently from the requirement to perform complementary/imitative actions.

The SMART-D software package (B|T|S|) was used to analyse data and provide a 3D reconstruction of the marker positions as a function of time. We recorded the kinematics of the entire blocks; then, during offline analyses, the times of participants' start button hand release and index-thumb contact times on the bottle were used to subdivide the kinematics recording with the aim of analysing only the reach-to-grasp phase (from start button hand release to index-thumb contact times) and not the movements performed by participants to return their hand to the starting position while waiting for the next trial.

To obtain specific information on the reaching component of the movement, we analysed wrist trajectory as indexed by the maximum peak of wrist height on the vertical plane (maxH). To obtain specific information on the grasping component of the movement, we analysed maxAp (that is, the maximum peak of index-thumb 3D Euclidean distance).

Behavioural or kinematics values that fell 2.5 s.d.'s above or below each individual mean for each experimental condition were excluded as outliers (on average, 0.7±0.08% of total (2.4±0.3 trials) in experiment 1, and 0.7±0.2% of total (1.6±0.5 trials) in experiment 2). At the group level, participants with an individual mean 2.5 s.d.'s above or below the group mean were excluded from the analyses; one participant in experiment 2 was an outlier on GAsynchr according to this criterion. Thus, she was excluded from the analyses and replaced (final sample 12 participants).

Each behavioural- and kinematics-dependent measure was then normalized on the individual grand mean and s.d. (*Z*-transformation) and entered in separate ANOVAs.

In experiment 1, the repeated measure ANOVAs had stimulation site (aIPS/vertex/sham) × action-type (complementary/imitative) × clip-type (correction/no-correction) × movement-type (power/precision grip) as within-subject factors (that is, 3 × 2 × 2 × 2 within-subject design). In experiment 2, the repeated measure ANOVAs had stimulation site (aIPS/vertex) × action-type (complementary/imitative) × clip-type (correction/no-correction) × movement-type (power/precision grip) as within-subject factors (that is, 2 × 2 × 2 × 2 within-subject design).

With regard to accuracy, we verified the absence of speed-accuracy trade-offs with GAsynchr by means of non-parametric tests on the condition of interests, that is, the site × action-type significant interaction. We calculated the individual difference between individual mean accuracy in complementary minus imitative action per each stimulation site and applied a Friedman ANOVA in experiment 1, aIPS (complementary–imitative) versus vPM (complementary–imitative) versus sham (complementary–imitative), and a Wilcoxon-matched pair test in experiment 2, aIPS (complementary–imitative) versus vertex (complementary–imitative). All tests of significance were based on an α-level of 0.05. When appropriate, *post hoc* tests were performed using the Newman–Keuls method.

## Additional information

**How to cite this article:** Sacheli, L.M. *et al.* Causative role of left aIPS in coding shared goals during human–avatar complementary joint actions. *Nat. Commun.* 6:7544 doi: 10.1038/ncomms8544 (2015).

## Supplementary Material

Supplementary Information Supplementary Figures 1 and 2

## Figures and Tables

**Table 1:** Significant effects from experiment 1 (*n*=14).

**Effect**	***F***	**df**	***P***	***η***^*2*^
*Grasping asynchrony*				
Main effect of action-type	11.88	1,13	0.004	0.477
Main effect of movement-type	8.46	1,13	0.012	0.394
Action-type × movement-type	35.81	1,13	<0.001	0.734
Action-type × clip-type × movement-type	89.37	1,13	<0.001	0.873
Site × action-type	***5.37***	***2,26***	***0.011***	***0.292***
				
*Reaction times*				
Action-type × clip-type	5.49	1,13	0.036	0.297
Site × action-type	***3.5***	***2,26***	***0.045***	***0.212***
Site × movement-type	***3.8***	***2,26***	***0.036***	***0.226***
				
*Movement times*				
Main effect of clip-type	255	1,13	<0.001	0.951
Action-type × movement-type	2,560	1,13	<0.001	0.995
Action-type × clip-type × movement-type	1,131	1,13	<0.001	0.989
				
*maxH*				
Main effect of clip-type	97.5	1,13	<0.001	0.882
Main effect of movement-type	139.1	1,13	<0.001	0.914
Action-type × clip-type	10.9	1,13	0.006	0.456
Action-type × movement-type	13.3	1,13	0.003	0.505
Clip-type × movement-type	105.8	1,13	<0.001	0.890
Action-type × clip-type × movement-type	10.9	1,13	0.006	0.456
				
*maxAp*				
Main effect of clip-type	350.4	1,13	<0.001	0.964
Main effect of movement-type	316	1,13	<0.001	0.960
Action-type × clip-type	16.1	1,13	<0.001	0.553
Clip-type × movement-type	118.4	1,13	<0.001	0.901
Clip-type × action-type × movement-type	19.9	1,13	<0.001	0.605

ANOVA, analysis of variance; maxAp, maximum grip aperture; maxH, maximum wrist height.

The table reports all significant effects showed by the repeated measures ANOVAs on *z*-scores of grasping asynchrony, reaction times, movement times and wrist and pre-shaping kinematics (maxH and maxAp), having stimulation site (aIPS/vertex/sham) × action-type (complementary/imitative) × clip-type (correction/no-correction) × movement-type (power/precision grip) as within-subject factors (see Methods section). In bold and italics are the significant interactions including the factor stimulation site described in the main text.

**Table 2:** Significant effects from experiment 2 (*n*=12).

**Effect**	***F***	**df**	***P***	***η***^*2*^
*Grasping asynchrony*				
Main effect of clip-type	14.69	1,11	0.003	0.572
Action-type × clip-type × movement-type	6.14	1,11	0.031	0.358
Site × action-type	***7.54***	***1,11***	***0.019***	***0.407***
				
*Reaction times*				
No significant effect	*—*	*—*	*—*	*—*
				
*Movement times*				
Main effect of clip-type	266.4	1,11	<0.001	0.960
Action-type × movement-type	1,264	1,11	<0.001	0.991
Action-type × clip-type × movement-type	62.94	1,11	<0.001	0.851
				
*maxH*				
Main effect of clip-type	297.9	1,11	<0.001	0.964
Main effect of movement-type	213.4	1,11	<0.001	0.950
Action-type × clip-type	13.2	1,11	0.004	0.546
Clip-type × movement-type	240.9	1,11	<0.001	0.956
Action-type × clip-type × movement-type	5.6	1,11	0.038	0.336
				
*maxAp*				
Main effect of clip-type	296.9	1,11	<0.001	0.964
Main effect of movement-type	269.1	1,11	<0.001	0.960
Action-type × clip-type	5.6	1,11	0.037	0.338
Clip-type × movement-type	175.7	1,11	<0.001	0.941
Clip-type × action-type × movement-type	11.6	1,11	0.006	0.512

ANOVA, analysis of variance; maxAp, maximum grip aperture; maxH, maximum wrist height.

The table reports all significant effects showed by the repeated measures ANOVAs on *z*-scores of grasping asynchrony, reaction times, movement times and wrist and pre-shaping kinematics (maxH and maxAp), having stimulation site (aIPS/vertex) × action-type (complementary/imitative) × clip-type (correction/no-correction) × movement-type (power/precision grip) as within-subject factors (see Methods section). In bold and italics are the significant interaction between action-type × stimulation site on grasping asynchrony described in the main text.
