# Supplementary material for: Causative role of left aIPS in coding shared goals during human–avatar complementary joint actions
Source: Nat Commun. 2015 Jul 8;6:7544. doi: 10.1038/ncomms8544 (PMC4510640; doi:10.1038/ncomms8544)
Supplement: Supplementary Information — Supplementary Figures 1 and 2 [file ncomms8544-s1.pdf]

Causative role of left aIPS in coding shared goals during human-avatar complementary joint actions

Lucia M Sacheli[1,2§*], Matteo Candidi[1,2§], Vanessa Era[1,2], Salvatore M Aglioti[1,2*]

Department of Psychology, University of Rome "Sapienza", I-00185, Italy, and IRCCS, Fondazione Santa Lucia, Rome, I-00179, Italy.

§ These authors contributed equally to this work.

* Corresponding authors.

Supplementary Figures

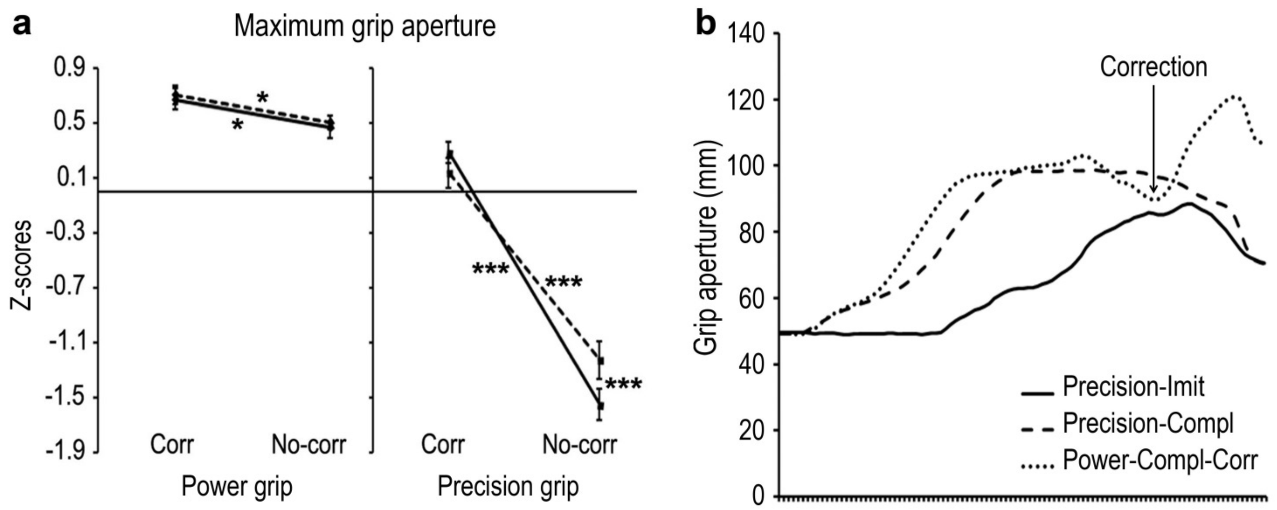

Supplementary Figure 1. Experiment 2 (n=12), data on maximum grip aperture (*maxAp*) kinematics. **(a)** The figure reports results from the significant Action-type x Clip-type x Movement-type interaction ($F(1,11) = 11.6$, $p = .006$) showed by the ANOVA *maxAp* (mean values). For the sake of simplicity, the significance of the comparison between Power and Precision grip in all conditions (all *p*s ≤ .001) is not explicitly reported. Note that significant effects are more relevant in Precision grip because the index-thumb distance reaches its maximum during Power grips (ceiling effect) thus masking the tendency to mimic the partner's precision grip. "Corr" = Corrections; "No-corr" = No-Corrections. Error bars indicate s.e.m. (***) $p < .001$, (*) $p < .05$. **(b)** Raw grip aperture profile recorded from a representative participant during an Imitative Precision grip (thick line), Complementary Precision grip (striped line) and during a Correction from Precision to Power grip (dotted line). The arrow indicates when the Correction occurred in the grip aperture profile (1.e., when the participant corrects her pre-shaping to a Power grip after having prepared a Precision grip). These data show that participants i) performed on-line corrections upon observation of the avatar's movement corrections, factually adapting to the avatar's movements, and ii) showed automatic mimicry of the avatar's movements during complementary actions, in line with previous studies on human-human interactions. Importantly, these effects were independent from cTBS stimulation, suggesting aIPS stimulation did not interfere with movement execution.

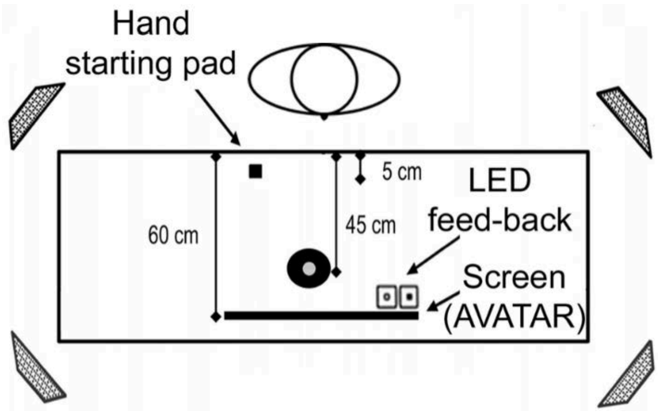

Supplementary Figure 2. Bird-view of the experimental set-up. Participants sat in front of the screen with their right hand placed on the start button and reached-to-grasp their bottle-shaped object (45 cm far from them) trying to be as synchronous as possible with the avatar's movement. A pair of green/red LED lights was placed next to the screen in order to provide feedback signals about participants' performance. Please note this figure has already been published as part of Figure 1 in [1].

Supplementary References

1. Sacheli, L.M. et al. Prejudiced interactions: implicit racial bias reduces predictive simulation during joint action with an out-group avatar. *Sci Rep*. **5,** 8507 (2015).
